# Genome-wide mapping of quantitative trait loci in admixed populations using mixed linear model and Bayesian multiple regression analysis

**DOI:** 10.1186/s12711-018-0402-1

**Published:** 2018-06-19

**Authors:** Ali Toosi, Rohan L. Fernando, Jack C. M. Dekkers

**Affiliations:** 1 Cobb-Vantress Inc., 4703 US HWY 412 E, Siloam Springs, AR 72761 USA; 2 Department of Animal Science, Iowa State University, Ames, IA 50010 USA

## Abstract

**Background:**

Population stratification and cryptic relationships have been the main sources of excessive false-positives and false-negatives in population-based association studies. Many methods have been developed to model these confounding factors and minimize their impact on the results of genome-wide association studies. In most of these methods, a two-stage approach is applied where: (1) methods are used to determine if there is a population structure in the sample dataset and (2) the effects of population structure are corrected either by modeling it or by running a separate analysis within each sub-population. The objective of this study was to evaluate the impact of population structure on the accuracy and power of genome-wide association studies using a Bayesian multiple regression method.

**Methods:**

We conducted a genome-wide association study in a stochastically simulated admixed population. The genome was composed of six chromosomes, each with 1000 markers. Fifteen segregating quantitative trait loci contributed to the genetic variation of a quantitative trait with heritability of 0.30. The impact of genetic relationships and breed composition (BC) on three analysis methods were evaluated: single marker simple regression (SMR), single marker mixed linear model (MLM) and Bayesian multiple-regression analysis (BMR). Each method was fitted with and without BC. Accuracy, power, false-positive rate and the positive predictive value of each method were calculated and used for comparison.

**Results:**

SMR and BMR, both without BC, were ranked as the worst and the best performing approaches, respectively. Our results showed that, while explicit modeling of genetic relationships and BC is essential for models SMR and MLM, BMR can disregard them and yet result in a higher power without compromising its false-positive rate.

**Conclusions:**

This study showed that the Bayesian multiple-regression analysis is robust to population structure and to relationships among study subjects and performs better than a single marker mixed linear model approach.

## Background

Like any other type of statistical association analysis, the purpose of a genetic association test is to establish an association between, or examine independence of, two variables: a trait of interest and a genetic marker [1, 2]. If the marker being tested is known to be a neutral locus without any known effects on DNA coding, then the linkage disequilibrium (LD) between the marker and a quantitative trait locus (QTL) affecting the trait could be a valid reason for the observed association [1]. The ideal condition in a genome-wide association study (GWAS) is that the covariance, and hence the LD, between a genetic marker and the trait of interest is high if, and only if, the marker itself is a causative mutation or is closely linked to a QTL [3]. This requires a panmictic population. Unfortunately, except in population genetics theory, this type of population probably does not exist [4, 5]. Widespread prevalence of non-random mating (e.g., assortative mating) in livestock and crop populations has resulted in complex patterns of population stratification (PS) and genetic relationships between members of the population [6–10]. Without properly accounting for these factors, GWAS could lead to spurious false-positives (FP) (markers declared as significant but not closely linked to a QTL) and false-negatives (FN) (markers closely linked to a QTL but not declared as significant) in such populations due to extensive LD between syntenic and non-syntenic loci [9–26]. Compared to simple monogenic traits, complex polygenic phenotypes are more vulnerable to elevated FP rates in GWAS, where the magnitude of signals from multiple QTL may be comparable to those resulting from PS [27].

Many approaches have been developed to account for PS and relatedness in a population-based GWAS, including genomic control (GC), structured association (SA), principal component analysis (PCA), mixed linear models (MLM) and multiple regression analysis.

Without a doubt, the GC method [28] is simple and fast and is even applicable to pooled DNA samples [29]. However, it has lower power than other methods, especially in samples with a complex pattern of relationship and extensive PS [9, 16, 30–37]. The SA approach [12, 13, 38–41], uses a set of null markers to infer PS information for each individual in the sample before conducting an association test [42]. Most popular SA methods assume that the ancestry of each individual is drawn from one or more discrete sub-populations (the so-called “islands” model) [33, 43], an assumption that is not supported by real data [see 44 for an example]. SA methods have been shown to be suboptimal in protecting against FP in commercial crop or model organism populations [9, 15, 18, 21, 25, 35, 36, 43, 45, 46].

The PCA method [47, 48] is fast, avoids assumptions on which the SA methods rely on and, unlike SA, is robust to the number of modeled principal components (PC) [49]. The idea of PC-adjustment for protecting against PS is valid under an island model but this might not be true for samples with complex PS. As a result, the method is only successful when applied to samples with mild PS [50, 51]. PCA may produce artefactual PC in the presence of outliers [52], of long-range LD on the genome [33, 34, 44, 53, 54] or of family structure or cryptic relatedness in the sample [33, 47]. The success of PC adjustment to control FP is conditional on whether sufficient numbers of PC are included in the model [33]. While inclusion of not enough PC may reduce the chance of controlling FP, inclusion of too many PC could undermine the power of the association study [29, 52]. On the one hand, pruning of markers has been suggested [33] and applied as an ad-hoc procedure for reducing the correlation between adjacent markers e.g., [55] before applying PCA. However, this could lead to loss of some subpopulation differences [20]. On the other hand, if some of the markers that are truly associated with the trait of interest fall in the pruned regions, then adjusting for PC is counterproductive [54]. Overall, there still is uncertainty on the proper method of PC adjustment and the optimal criteria for selecting PC to be retained [56, 57].

One might identify the above approaches as two-step methods for correcting for PS. They can eliminate the true association signals whenever the strength of association due to PS is comparable to that of a QTL. Simultaneous inference of PS and testing for association has the advantage of being able to separate the true and false signals from each other [3, 15]. Yu et al. [9] used a set of unidentified markers to detect population structure (Q) and familial relationship (K) in a maize sample dataset. They fit both Q and K into a mixed linear model to account for multiple levels of relationship in the dataset. This method was shown to perform better than other methods in controlling FP and FN rates [9, 15, 21, 25, 33, 35, 36, 45, 58, 59]. However, the MLM approach is computationally expensive if applied to a large dataset [58] and its success in finding associations may depend on the minor allele frequency (MAF) of the markers. In fact, with MLM strong phenotypic associations are easier to detect when the MAF is low [60]. In short MLM, and the other above-mentioned approaches, might not be suitable when applied to complex traits controlled by several large-effect loci [61].

In principle, PS simply can be adjusted for by including a set of ancestry-informative or null markers as covariates in the model. These markers or a function of them can effectively serve as proxies for the underlying PS [1, 16, 29, 32, 62]. Valdar et al. [63] suggested modeling PS explicitly in a multi-marker association analysis (MMA) framework. By comparing single-marker association (SMA) analysis with the MMA model, they showed that family structure should be considered in the SMA model to obtain reasonable power, whereas the MMA model could safely ignore this effect without compromising its power. However, when the sample was highly structured, the MMA model suffered from high FP [63]. Pikkuhookana and Sillanpaa [64] compared the impact of including versus ignoring pedigree relationships in a Bayesian multiple regression (BMR) model using simulated and real data. They found that Bayesian MMA analysis without correction for relationship was capable of self-correcting for residual dependencies and did not produce spurious associations. In a comprehensive simulation study, Setakis et al. [16] used logistic regression in a SMA study of a binary trait and were also able to account for PS without explicitly modeling it.

In recent years, genomic selection (GS) [65] has shown promising results for predicting breeding values (BV) of selection candidates [66]. In this approach, the effects of markers across the genome are estimated first in a reference population (training dataset) and then are used to predict the BV of individuals in an independent dataset (validation dataset). Simulation studies of GS in multi-breed admixed populations [67–69] have shown that the estimated effects of markers in such samples might accurately predict the BV of purebred animals in a validation dataset, provided that marker density is sufficient to capture the shared ancestral LD across breeds. Thomasen et al. [70] studied population structure in a Danish Jersey population composed of subgroups of animals that originated from Danish or United State Jersey populations and showed that a model that explicitly accounts for breed origin, does not improve genomic predictions compared to a model that ignores breed origin. These results suggest that the performance of QTL mapping in an admixed population, using a BMR approach with high-density markers, may not be hampered by the spurious FP when BC and relatedness have not been explicitly accounted for. Therefore, the purpose of this study was to evaluate the performance of genome-wide QTL mapping in a highly structured admixed population typical of animal and plant breeding datasets using the BMR and to compare that with the performance of a MLM approach, which has been the method of choice for many recent GWAS.

## Methods

### Population

A base population of unrelated individuals was stochastically simulated and used as described below to create four pure breeds, and admixed and crossbred populations based on these breeds. To generate LD, the base population was randomly mated for 1000 generations, with an effective size (N_e_) of 1000. To simulate the four purebred populations (referred to as breeds A, B, C and D, hereafter), at generation 1001 four independent random samples of 100 animals were drawn from the base population and each was randomly mated for another 50 generations, with an N_e_ of 100. A previous study [69] showed that this setting is successful for creating genetically diversified breeds.

In generation 1051, pure breed population sizes were increased to N = 1000. Each population was composed of 50 half-sib families with an average size of 20 offspring per family, created by random mating of sires and dams from the previous generation. No attempt was made to keep family sizes equal. These breeds were then crossed to create (AB), (AB)A, (AB)C and (AB)(CD) populations. This resulted in eight different populations (including the four pure breeds) of size 1000. Finally, a random sample of 1000 individuals was drawn from the pool of all populations and used as the admixed population. The pure breed A and admixed datasets at generation 1053 (referred to as the training generation hereafter) were used as the resource populations for QTL mapping. PCA based on whole-genome marker genotypes was used to verify the population structure in the simulated admixed dataset.

### Genome

A genome of size 600 cM composed of six chromosomes that each had 5000 equally spaced markers was simulated. Markers were bi-allelic, with starting allele frequencies of 0.5 and a reversible random mutation rate of 2.5 × 10^−5^. A binomial map function was used to simulate recombination and interference was allowed for by setting the maximum number of uniformly and independently distributed crossovers on the chromosome to 4 [71]. At generation 1053, 1000 markers were selected from the remaining segregating markers for each chromosome.

### Phenotypes

In the training generation, 15 segregating markers (MAF > 0.02) that were closest to certain positions on chromosomes 1 to 3 (Table 1) were chosen to represent the QTL with an assigned effect. Chromosomes 4 to 6 (referred herein to as null chromosomes) did not contribute to the simulated phenotype. Markers that were assigned to be QTL were removed from the marker panel before association analysis. To keep the genetic variance constant across the simulated datasets, the allele substitution effects of the QTL were standardized such that each QTL explained a predefined percentage of the total genetic variance in the admixed population (Table 1). Only additive effects were simulated. With equal probability, allele substitution effects were assigned to be negative or positive. Then, the scaled QTL effects were summed over all QTL for each individual to compute an individual’s true BV. Finally, a standard normal deviate was added to each true BV to provide the phenotype of an individual for a quantitative trait with heritability 0.30. The simulation was conducted for 32 different QTL minor allele frequencies. For each of these scenarios (which we refer to hereafter as a dataset), we replicated the simulation 20 times, allowing some variation in the QTL position and the surrounding marker genotypes.

**Table 1:** Simulated QTL positions (cM) and effects

Chromosome	QTL position	% of phenotypic variance explained by QTL
1	60	0.01
1	61	0.01
1	95	0.01
2	121	0.01
2	125	0.01
2	160	0.01
3	205	0.01
3	215	0.01
3	225	0.01
3	240	0.01
1	75	0.03
2	120	0.03
2	180	0.03
3	270	0.03
1	15	0.06

### Association mapping methods

The following models were used to analyze the simulated datasets.

#### Single marker association analysis (SMA)

Simple regression analysis was used to examine association of each marker’s genotype with each individual’s phenotypic value. Markers were fitted one-at-a-time using the following linear model:

1 $${\mathbf{y}} = {\mathbf{1}}\mu + {\mathbf{w}}a + {\mathbf{e}},$$

where ${\mathbf{y}}$ is the vector of phenotypic values of size n, ${\mathbf{1}}$ is a vector of ones of length n, $\mu$ is the population mean, ${\mathbf{w}}$ is a vector of the genotypic values at a marker locus (0, 1 or 2; number of copies of an arbitrary allele at the marker being tested), $a$ is the fixed allele substitution effect and ${\mathbf{e}}$ is the vector of random residual errors. The model improperly assumes that ${\mathbf{y}} \sim N({\mathbf{1}}\mu + {\mathbf{w}}a,{\mathbf{I}}\sigma_{e}^{2} )$. We applied this model only for the sake of comparison. The analysis was done using the PLINK software package with its *assoc* option [72].

#### Single marker association analysis with breed composition (SMA_BC_)

The second model explicitly considered BC in the admixed population:

2 $${\mathbf{y}} = {\mathbf{1}}\mu + {\mathbf{X}}{\varvec{\upbeta}} + {\mathbf{w}}a + {\mathbf{e}},$$

where ${\mathbf{X}}$ is the n × q incidence matrix relating observations to BC and ${\varvec{\upbeta}}$ is a fixed vector of BC. The true BC of each individual was assumed known without error. Furthermore, it is assumed that ${\mathbf{y}} \sim N({\mathbf{1}}\mu + {\mathbf{X}}{\varvec{\upbeta}} + {\mathbf{w}}a, {\mathbf{I}}\sigma_{e}^{2} )$. All other parameters and assumptions were the same as in Model (1). *ASReml* [73] was used for analysis. The Wald test, as implemented in the software, was used for significance tests of the marker-trait association and BC effects. Both the SMA and SMA_BC_ models are inadequate in that they do not account for genetic relationships in the population. As a result, the assumptions under which the null hypothesis is being tested might not be valid.

#### Single marker mixed linear model (MLM)

Conventional mixed model analysis, fitting one marker at a time, was applied using the following model:

3 $${\mathbf{y}} = {\mathbf{1}}\mu + {\mathbf{Zu}} + {\mathbf{w}}a + {\mathbf{e}},$$

where ${\mathbf{Z}}$ is the incidence matrix relating observations to the corresponding random effect and ${\mathbf{u}}$ is the vector of random additive genetic effects or BV. It was assumed that ${\mathbf{y}} \sim N({\mathbf{1}}\mu + {\mathbf{w}}a,{\mathbf{ZGZ^{\prime}}} + {\mathbf{R}})$ and ${\mathbf{u}}|\sigma_{u}^{2} \sim N({\mathbf{0}},{\mathbf{G}})$, where ${\mathbf{G}} = {\mathbf{A}}\sigma_{u}^{2}$ and ${\mathbf{R}} = {\mathbf{I}}\sigma_{e}^{2}$. Here ${\mathbf{A}}$ is the matrix of additive genetic relationships, where $a_{ij}$ is twice the coefficient of coancestry between individuals $i$ and $j$, and $\sigma_{u}^{2}$ is the additive genetic variance. Other parameters were as introduced before. *ASReml* was used for analysis and testing of marker effect was based on the Wald test implemented in the software.

#### Single marker mixed linear model with breed composition (MLM_BC_)

This model was similar to Model (3) except that BC was also included as a fixed effect factor.

#### Bayesian multiple regression (BMR)

Stochastic search variable selection is a hierarchical Bayesian model that stochastically searches for ‘promising’ subsets of predictors [74]. Properties of such models have been discussed in detail elsewhere [75, 76]. We used the BayesCπ method of Habier et al. [77].

4 $${\mathbf{y}} = {\mathbf{1}}\mu + \mathop \sum \limits_{k} \gamma_{k} {\mathbf{w}}_{k} \alpha_{k} + {\mathbf{e}},$$

where ${\mathbf{w}}_{k}$ is a column vector of marker genotypes at locus $k$ and $\gamma_{k}$ is a latent 0/1 variable showing absence or presence of marker $k$ in the model. Here $\alpha_{k}$ is the random substitution effect of marker $k$ and is assumed a priori independently distributed as:

$$\left. {\alpha_{k} } \right|\pi , \sigma_{{\alpha_{k} }}^{2} = \left\{ {\begin{array}{*{20}l} 0 \hfill &\quad {\text{with probability}}\; \pi \hfill \\ { \sim N\left( {0,\sigma_{{\alpha_{k} }}^{2} } \right) } \hfill &\quad {{\text{with probability}} \;\left( {1 - \pi } \right)} \hfill \\ \end{array} ,} \right.$$

where $\sigma_{k}^{2}$ are assumed a priori independently and identically distributed (*iid*) scaled inverted Chi square variables with scale and shape parameters of $S_{\alpha }^{2}$ and $\nu_{\alpha }$, respectively. Note that $\pi$ determines the sparsity of the model. Residuals were assumed *iid* and $e \sim N(0,\sigma_{e}^{2} )$. Furthermore, it was assumed a priori that $\sigma_{e}^{2}$ follows a scaled inverted Chi square distribution with parameters $S_{e}^{2}$ and $\nu_{e}$, respectively. A deterministic approach was used to find the hyper parameters of the prior distribution of $\sigma_{{\alpha_{k} }}^{2}$, as described in Habier et al. [77].

A Gibbs sampler was used to generate a Markov chain Monte Carlo (MCMC) chain of 100,000 samples with a burn-in period of length 10,000. Convergence of the chain was examined using the R software package CODA [78] and visual inspection of the chain plots. The posterior inclusion probability (PIP) e.g. [75] of a marker, $\Pr (\gamma_{k} = 1 |y)$, was calculated as the average of all post burn-in values of $\gamma_{k}$.

#### Bayesian multiple-regression with breed composition (BMR_BC_)

This model was similar to model [5] except that BC was also included as a fixed effect.

### Estimation of significance thresholds

To estimate the thresholds required for hypothesis testing, each of the null chromosomes was divided into non-overlapping bins of 40 markers (± 2 cM). The average LD, measured as R^2^, between consecutive loci 1 and 2 cM apart was about 5.5 and 3.5%, respectively. Generally speaking, the ± 2 cM interval is the preferred precision of interest for QTL detection or efficient implementation of QTL information in marker-assisted selection (e.g., [79]) and hence it was used here. For each bin, the minimum P-values for the non-Bayesian approaches (or the maximum PIP value for the Bayesian approaches) were recorded for each replicate. For each dataset that comprised 20 replicates, the sets of these values for all bins on the null chromosomes were combined and used to determine the 5% (or the 95% for the Bayesian approaches) *quantile* of their distribution using the *quantile* function in R [80]. We refer to this approach based on the empirical distribution of P- or PIP-values on the null chromosomes [81], as the null-chromosome (NCHR) method of finding thresholds. The method might be considered comparable to a permutation test where the phenotypes are reshuffled to disrupt the marker-trait associations, as explained in Xu [82]. This method was used here to make the comparison between the non-Bayesian and the Bayesian approaches feasible, as suggested in Sahanaet al. [81]. For the non-Bayesian approaches, the SLIDE method of Han et al. [83] was used as an alternative for finding thresholds. SLIDE first estimates the effective number of tests ($M_{eff}$) using a sliding window Monte-Carlo approach. Then, a Bonferroni threshold can be calculated by dividing the nominal P-values by the $M_{eff}$. The sliding window MCMC approach approximates the asymptotic multivariate normal distribution of the test statistic and accounts for all correlations among markers within a sliding window. We ran SLIDE with a window size of 40 markers and applied 100 k cycles of an MCMC chain. The program estimated the $M_{eff}$ and then this number was used as the actual number of markers (rather than the 5985 markers that were actually on the panel) for calculating the Bonferroni adjusted P-values. Quantile–Quantile plots were used to characterize the extent to which the distribution of P-values on the null chromosomes deviated from their expected distributions for the different association analysis methods.

### QTL detection and power calculation

For each replicate of a dataset, a QTL was declared detected if any of the markers within an interval of ± 2 cM of the QTL (40 markers in total) had a P-value smaller than the 5% threshold P-value (for the non-Bayesian analysis), or a PIP value larger than the 95% threshold PIP value (for the Bayesian analysis). Power was defined as the proportion of times that a QTL was detected out of 20 replicates in that dataset.

#### False positive rates (FPR), accuracy, and positive prediction values (PPV)

Excluding the ± 2 cM intervals harboring the QTL, the remaining parts of chromosomes 1, 2 and 3 were divided into 4-cM long segments, as intervals where the null hypothesis was correct. If a marker was declared significant in any of these intervals, it was regarded as a false-positive. FPR was the proportion of false-positives across the genome and then averaged over all replicates of a single dataset. Comparing power of methods that have different FPR could be misleading because positive results might be due to PS as well. Therefore, two other measurements, accuracy and PPV were also used to evaluate the performance of the different models [35]. Positive and negative results falling in the H_1_ regions (intervals where H_0_ is false) were counted as true-positives (TP) and false-negatives (FN), respectively. Similarly, positive and negative results in H_0_ regions were counted as false-positives (FP) and true-negatives (TN). Then, accuracy and PPV were computed as:

$$Accuracy = \frac{TP + TN}{TP + TN + FP + FN},$$

$$PPV = \frac{TP}{TP + FP}.$$

All performance measures, i.e., accuracy, power, FPR and PPV, were calculated on a per dataset basis and then averaged across all datasets. All QTL sizes showed a similar trend in the above performance measures, hence instead of calculating the performance measures for each QTL size, we report averages across all QTL in a dataset.

## Results

### Population stratification

**Fig. 1:**
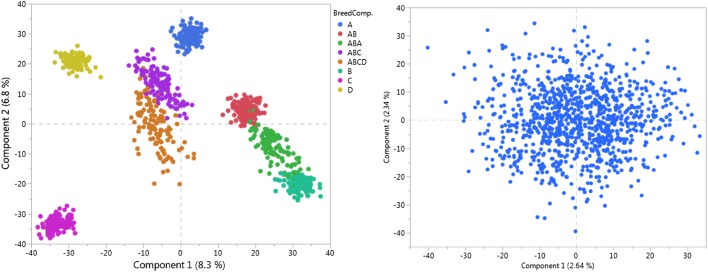
Scatter plots of the first two principal components of the genome-wide markers in the admixed (left) and the purebred (right) populations. Numbers in brackets show the percentage of variances explained by corresponding PC. Different colors represent various breed compositions

PCA of the marker data for the purebred and admixed populations revealed distinct clusters of related animals within the admixed population, in contrast to the purebred population (Fig. 1). In addition, the effect of BC was highly significant in all non-Bayesian analyses where this term was included in the model. This makes proper modeling of population structure compulsory in order to conserve the type I error rate.

### Distribution of P-values on the null chromosomes

**Fig. 2:**
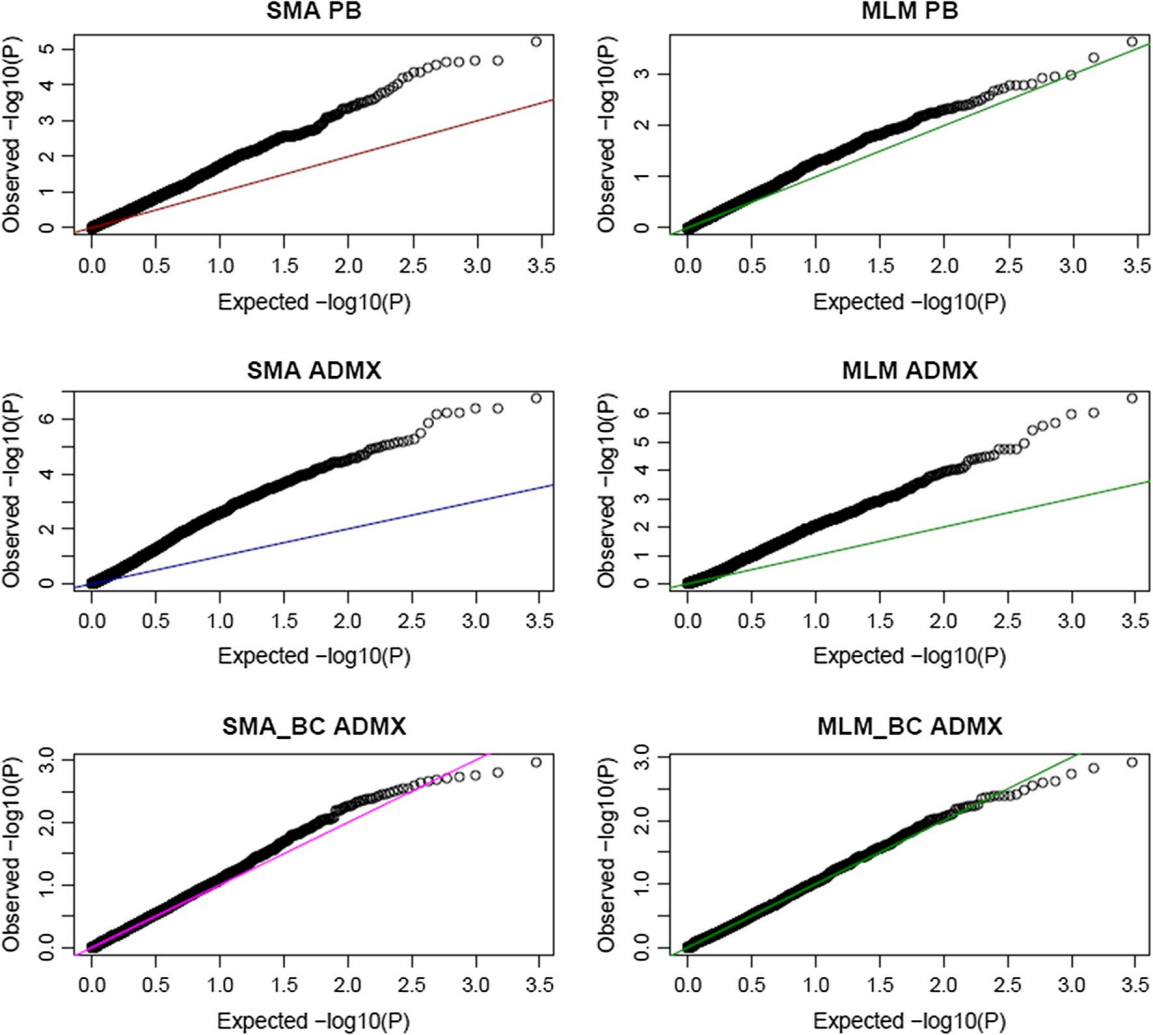
Q–Q plots of the observed distribution of − log10(P-values) on the null chromosomes, with different analysis approaches, versus their expected distribution. *PB* purebred population, *ADMX* admixed population, *SMA* single marker association, *SMA_BC* SMA with breed composition, *MLM* mixed linear model association, *MLM_BC* MLM with breed composition

Examination of the Q–Q plots of P-values of the markers on null chromosomes showed spurious FP in the association analyses of both the purebred and the admixed populations when relationships and/or BC were not modeled properly (Fig. 2).

### Single-marker association analyses

Results of the SMA and MLM analyses of the ADMX population are in Tables 2 and 3. As expected, the SMA model had the lowest accuracy and PPV and the highest FPR among the four tested models. On the one hand, with the NCHR method of finding thresholds (Table 2), modeling BC increased power of QTL detection and PPV by 60 and 20 to 30%, respectively, but at the cost of a nearly 20% inflation of FPR. With NCHR, the accuracy of QTL detection was the same for all methods used for analysis. On the other hand, modeling BC dramatically improved accuracy, FPR and PPV when the SLIDE method was used for finding thresholds (Table 3); the accuracies of models accounting for BC were 30 to 60% higher than those that did not, improved PPV by 300% but resulted in loss of power by more than 50%.

**Table 2:** Accuracy, power, false positive rate and positive predictive value (PPV) for the SMA and MLM analyses with the NCHR method of finding thresholds in the ADMX population

	SMA	SMA_BC_	MLM	MLM_BC_
Accuracy	0.86 (0.003)	0.87 (0.005)	0.86 (0.005)	0.87 (0.004)
Power	0.40 (0.027)	0.63 (0.038)	0.40 (0.049)	0.64 (0.035)
False positive rate	0.08 (0.003)	0.10 (0.007)	0.08 (0.006)	0.11 (0.006)
PPV	0.34 (0.016)	0.43 (0.014)	0.36 (0.031)	0.43 (0.012)

Numbers in brackets are SE of means

**Table 3:** Accuracy, power, false positive rate and positive predictive value (PPV) for SMA and MLM analyses with the SLIDE method of finding thresholds in the ADMX population

	SMA	SMA_BC_	MLM	MLM_BC_
Accuracy	0.58 (0.047)	0.92 (0.003)	0.69 (0.078)	0.92 (0.002)
Power	0.72 (0.034)	0.30 (0.026)	0.63 (0.063)	0.27 (0.021)
False positive rate	0.44 (0.056)	0.007 (0.001)	0.30 (0.096)	0.004 (0.001)
PPV	0.25 (0.030)	0.85 (0.018)	0.34 (0.057)	0.90 (0.017)

Numbers in brackets are SE of means

Table 4, shows the results of the Bayesian association analysis in the ADMX population. With a density of 10 markers per cM, adding BC as a fixed effect into the model reduced both power and FPR by 11 and 13%, respectively. However, accuracy and PPV of QTL detection remained nearly unchanged.

**Table 4:** Accuracy, power, false positive rate and positive predictive value (PPV) for BMR analysis in the ADMX population

	BMR	BMR_BC_
Accuracy	0.89 (0.002)	0.89 (0.002)
Power	0.65 (0.016)	0.58 (0.017)
False positive rate	0.08 (0.001)	0.07 (0.001)
PPV	0.51 (0.007)	0.51 (0.008)

Numbers in brackets are SE of means

## Discussion

In this simulation study, we compared three methods of GWAS in an admixed population: single marker simple regression, single marker mixed model and Bayesian multiple regression models, with- and without- fitting breed composition.

### Effect of breed composition

The PCA showed that there was a distinct PS in the ADMX dataset. In a study by Toosi, Fernando and Dekkers [69], who simulated different breeds using the same scenario as described here, the genetic distance between breeds was nearly 24% based on Wright’s *F*_ST_ statistic. PS will be a source of spurious associations if both allele frequencies and mean phenotypic values differ between the sub-populations [84]. The effect of BC was highly significant (P < 0.001) in both SMA and MLM analyses (data not shown).

### Single-marker association models

#### Association tests based on the null chromosomes

The PB dataset showed spurious FP when the pedigree relationships in the population were ignored, as expected (Fig. 2). Unequal relatedness within a sample can result in increased FP rates in two ways: first, regions where QTL reside may be co-inherited with regions completely devoid of QTL [85] and second, genotype correlations within larger families can have a larger impact on the association results compared to the smaller families [86]. Kennedy et al. [87] showed that for both randomly mated and selected populations with complex pedigrees, the MLM approach provides unbiased estimates and exact tests of associations, whereas the ordinary least squares method does not. If dependencies among study subjects are not accounted for, many statistical tests of association are not strictly valid [88].

In the PB dataset, the MLM approach did control FPR at the nominal level on the null chromosomes, but it failed in the ADMX dataset without fitting BC. In this situation, any marker that has different allele frequencies between breeds shows association with the phenotype under study. The extent of FPR is a function of the extent to which the population is structured and not accounted for [15]. Therefore, for the highly divergent breeds simulated in our study, modeling BC was necessary for controlling FPR.

In a GWAS of a massively structured population consisting of 1800 bulls of the German Fleckvieh breed, Pausch et al. [55] applied the same SMA model as we did here and observed extensive significant association signals, possibly due to the variation of the relatedness between and within the families in the sample. Likewise, Wang et al. [36] conducted a GWAS of several morphological and agronomic traits in a highly structured population of barley cultivars and compared different PS correction methods. When they used a similar naïve SMA model, an excessive number of significant associations were found. In their study, MLM that incorporated kinship (K) [9] was superior to GC, SA and stepwise regression [16] in controlling FP rate and yielded higher power [36].

#### Association tests based on the SLIDE method

The changes in the performance of SMA models with and without fitting BC were most evident with the SLIDE method of finding thresholds (Table 3). On the one hand while modeling of BC improved the power of QTL detection with the NCHR method, this was not the case when SLIDE method was applied. On the other hand, for the SMA model, the FPR dropped dramatically when BC was fitted. This agrees with the result of Iwata et al. [17] who made a similar comparison. However, care must be taken when comparing the power of two methods that have different FPR, since positive results could be due to both true QTL signals and PS [35]. This is evidenced by the high FPR of models that did not fit BC (compared to those fitting it), when the SLIDE method was used for hypothesis testing. Furthermore, modeling BC sharply improved both the accuracy and the PPV of QTL detection. The SMA_BC_ models performed similar to the MLM_BC_, although the SMA_BC_ did not fully account for the kinship in the sample. It is possible that correcting for the PS has indirectly corrected some of the pedigree relationships between individuals in the sample and as a result, there were fewer spurious associations [45].

While there were no differences between accuracies of the SMA and MLM or the SMA and the SMA_BC_ when the NCHR method was used (Table 2), there were noticeable differences in these accuracies when the SLIDE method was applied. As an example for the SMA and SMA_BC_ methods, consider their accuracy (0.58 and 0.92, respectively), power (0.72 and 0.30) and FPR (0.44 and 0.007). It is evident from these results that many of the significant results of the SMA are false positives. Also, the difference between the accuracies of the SMA and SMA_BC_ implies that modeling BC has dramatically increased the number of TN. However, preventing the confounding effect of PS by explicitly modeling it, comes at a cost of more FN [22, 60, 89, 90]. Adjusting for PS may cancel out the effect of QTL that contribute to phenotypic differences between breeds [49]. Anderson et al. [91] conducted a GWAS on 32 lines of European inbred maize with different line origins. Comparing a model that adjusted for line origin versus one that did not, they showed that several true QTL remained undetected when line origin was accounted for, because these polymorphisms were confounded with line origin. This confounding is especially important for traits that have experienced adaptive selection and thus their variation may coincide with PS [22, 92].

In our study, fitting BC resulted in a considerable drop of power of QTL detection when the SLIDE method was used, but this was compensated for by a significant drop in FPR. Further inspection showed that in most instances, the smallest QTL were missed. This agrees with the findings of Iwata et al. [93], who showed that smaller QTL have larger FN rates.

To control family-wise type I error rate, SMA requires methods like Bonferroni correction for multiple testing. Such adjustments are usually too conservative, especially in a large scale SMA with extensive LD between linked markers, and thus they may cause true associations to be missed [26, 94–96]. That is why for most complex polygenic traits, SMA only detects a very small proportion of genetic variants [97, 98].

#### Multi-marker association tests

Comparison of the performance of the BMR models with and without fitting BC (Table 4) indicates that in the MMA framework, explicit modeling of PS might be unnecessary. The BMR model performed much better than the MLM and MLM_BC_. While modeling of BC in the MLM approach improved power of association (Table 2), it resulted in loss of power when the BMR was used. As expected, the FPR of the MMA methods were lower than the FPR of the SMA methods (Table 3). One major concern with SMA is that it ignores the information that is contained in the joint distribution of all markers [32, 96, 99]. A marker’s marginal effect might be different from its effect when it is considered jointly with some other markers. The BMR approach had the capability of model selection and hence it could decide whether to add or skip adding a marker to the set of pre-existing markers in the model. This function yields lower FPR over a SMA model. On the MMA framework, once the marker with the strongest marginal correlation with the phenotype is in the model, other markers that are in LD with this marker but that do not provide additional information about the phenotype are automatically discarded [26, 100, 101]. In addition, MMA analysis improves performance over SMA tests, first because a weak signal may be more apparent when other QTL are already accounted for, and second because a false signal may be weakened by inclusion of a stronger signal from a real QTL in the model [97].

#### Modeling of PS

Atwell et al. [21], in a GWAS of more than 100 phenotypes in inbred lines of *Arabidopsis thaliana*, showed that GWA yields unambiguous results for monogenic characters regardless of whether they corrected for PS or not. The authors concluded that the reason for this result was not that there were no confounding effects but because the true signals were showing the strongest associations. Therefore, they suggested that the problem of confounding due to PS in GWAS of complex traits might be better explained as a model misspecification, i.e., modeling a polygenic trait using a SMA that ignores the multi-factorial background of the trait. Not only SMA models result in spurious FP across the genome but they may also find the strongest associations on chromosomes that are completely devoid of QTL [3]. Therefore, Platt et al. [3] suggested that the real goal of GWAS in controlling PS effects should be to account for the confounding effects of multiple QTL, rather than modeling of PS per se.

Our results agree with Setakis et al. [16], Iwata et al. [17], Iwata et al. [93], Pikkuhookana and Sillanpaa [64], Karkkainen and Sillanpaa [50] and Valdar et al. [63], who demonstrated that unlike SMA models, MMA models are able to self-correct for family structure. Iwata et al. [17] proposed a Bayesian MMA for an empirical GWA in a rice germplasm collection. Their analysis of simulated data based on real marker genotypes revealed that their MMA could more successfully conserve both FP and FN compared to SMA models. In a GWAS of a highly structured population of barley cultivars, Wang et al. [36] compared the performance of the **Q** + **K** model with the **K**-only model, and with the GC, SA and PCA models. The **K**-only model outperformed all the other rivals. With high-density marker data, the **K** matrix contains all information on PS and hence the explicit modeling of PS might not be necessary [33, 102]. Apparently when marker density is sufficient, each marker might capture a part of the effects of kinship and PS, and as a result their overall effects are faded. This agrees with Sillanpää [103], who argued that in MMA models variable selection is done simultaneously with the estimation of effects and thus, the large number of markers considered jointly might account for many types of variations.

Gu et al. [26], applied a modified forward multiple regression (MFMR) approach based on maximum order statistics in an empirical GWAS. Their simulation was based on a 115 k Affymetrix single nucleotide polymorphism (SNP) panel and a dataset that was mainly composed of Caucasian, Black, and Hispanic races. They picked up three independent SNPs that were significantly correlated with race as QTL surrogates. When comparing the results of the SMA and MFMR analyses, they showed that the FPR of the MFMR approach was not affected by PS. This implies that once the QTL that is correlated with PS is included in the multiple regression model, the effect of PS has been accounted for [26]. Likewise, Pikkuhookana and Sillanpaa [64], who used a BMR model for a clinical QTL study in a sample with family structure, showed that regardless of having a correction term for PS in the model, the MMA fits a few extra markers with small effects. As a result, the MMA model was able to conserve both FP and FN rates. Another interesting finding in Gu et al. [26] that agrees with our result, was that fitting PS in the MFMR model reduced power without changing the FPR. Whenever attempting to control FPR in a GWAS, some FN are inevitable [18, 60, 89]. If the distribution of a QTL is highly correlated with PS, the effect of the allele may be absorbed in the PS effects and the QTL will be obscured [18, 22, 89, 94, 104, 105].

#### Implication of multi-population GWAS

Using a multi-population sample the detection of QTL that cause between-population differences is possible. A combined analysis of data from several populations takes advantage of the between-population genetic variability and hence is more powerful than single-population association study [24, 99, 106, 107]. A pooled sample of several breeds, for example, has potentially more informative recombination events and shorter haplotype lengths due to narrower LD distances across breeds [69, 99, 108].

As we showed in this study, and in agreement with studies of Gu et al. [26], Wurschum et al. [109] and Zhao et al. [18], explicit accounting for PS results in some FN. In fact, any method that effectively eliminates confounding due to PS will also effectively remove QTL that are highly correlated with PS [18]. This might be more of a problem with QTL with more subtle effects that are typical of complex traits and with the small sample sizes usually available for GWAS in animal breeding. While we showed here that the BMR method is capable of reducing FN due to implicit modeling of PS, the BMR also has the advantage of a lower FN compared to SMA that apply highly conservative multiple-test correction methods such as Bonferroni to their association results.

Teo et al. [27] showed the presence of opposing LD between populations, i.e., differences in the LD phase between a marker and a QTL across populations, can have a negative impact on the power of case-control or family-trio association studies. Fitting all markers simultaneously might overcome this problem. It is unlikely that all markers that are in LD with a specific QTL have a different LD phase with the QTL across populations, especially if they are close enough to the QTL. In a multi-population sample, markers in strong LD with QTL tend to be less distant to the QTL compared to that in a single-population sample [69]. In contrast, the SMA models that use GC, SA or PCA to control for the confounding effects of PS, might not be able to correct for the LD differences that reduce power in a multi-population association study [27]. Several studies have shown that leveraging the LD differences across populations—by conducting GWAS in a multi-population sample—may amplify the signal of QTL, because markers that are strongly linked to a QTL in one population may not be even segregating in another population [95, 106, 110].

As genomic selection approaches are gaining popularity, some recent GWAS in animal breeding dealing with multi-breed datasets have used methods similar to the BMR method we used here [111, 112]. In these studies, BC was added as a fixed effect in the model. If BC is confounded with some contemporary group effects (e.g., slaughter date or geographical region) that are not already accounted for, adding it as a fixed effect in the BMR model might be necessary. This will suppress association signals that are due to the correlation of the phenotype with the contemporary group effect and thus reduces FP. However, if BC is not confounded with any other effect and a MMA model such as that used here is applied, then the cost of implicit accounting for PS might be an increase of FN.

## Conclusions

In conclusion, our results show the superiority of MMA models over SMA models. More specifically, our study confirms that MMA models are capable of automatically accounting for the confounding effects of kinship and population structure in GWAS, without compromising the power of QTL detection.
